# Hydrogen bond density and glass-transition temperature govern gelatinization and gel rheology in cereal and tuber starches

**DOI:** 10.1016/j.crfs.2025.101101

**Published:** 2025-05-29

**Authors:** Stefano Renzetti, Jolanda Henket, Eric Raaijmakers, Irene van den Hoek, Ruud van der Sman

**Affiliations:** Wageningen Food and Biobased Research, Wageningen University & Research, Bornse Weilanden 9, 6700, AA Wageningen, the Netherlands

## Abstract

The gelatinization behaviour, pasting properties, and rheology of potato, cassava, rice, wheat, and waxy corn starches were studied. Gelatinization behaviour was examined at different starch-to-water ratios using differential scanning calorimetry to construct state diagrams of melting. The obtained onset, peak, and end temperatures of gelatinization were described using the Flory-Huggins theory for biopolymer melting. The validity of the obtained diagrams was tested against literature data, including gelatinization in different sugar solutions for all starches. Water-sugar mixtures were treated as a single solvent by considering the volumetric density of hydrogen bonds in the sugar solutions ($\Phi_{w,eff}$). All literature data collapsed into the melting diagrams, except for T_onset_ at high sugar concentrations, due to phase separation between starch-rich and sugar-rich phases. The pasting properties and the rheology of freshly prepared starch gels were analyzed at three concentrations (5, 8, and 11 % w/w) in water, revealing differences among the starches as a function of concentration. However, G′ and G″ values obtained from frequency sweeps scaled with the computed *T*_*g*_*/T*, based on amylose concentrations. Cereal and tuber starches exhibited distinct relationships with *T*_*g*_*/T*. Notably, literature data collected under similar experimental conditions aligned with the scaling observed in this study, despite differences in ingredient sourcing. Overall, this study provides insights into the physicochemical principles governing gelatinization and rheological behaviour in starches from diverse botanical sources. The findings offer a universally applicable understanding that can aid in designing cereal- and starch-based food formulations.

## Highlights

• State diagrams for melting of cereal and tuber starches were constructed.
• Melting described also in presence of sugars using the density of hydroxyl groups in the solvent.
• Rheology of starch gels after short storage described by *T*_*g*_*/T* based on amylose content.
• Different scaling observed between cereals and tubers.
• Literature data collapsed with the data of this study.

## Graphical abstract

**Figure:**
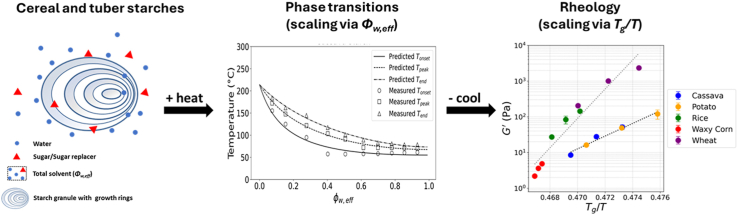

## 1 Introduction

Starch is a key macromolecule in many food products, significantly influencing their processing and nutritional properties. Starch interactions with water, sugars, and other polysaccharides are particularly important in food science. Naturally, starch exists as semi-crystalline, water-insoluble granules composed of two polysaccharides: amylose and amylopectin (Copeland et al., 2009; Tester et al., 2004). These form organized blocklets, creating a lamellar arrangement with alternating crystalline and amorphous layers (Donald, 2004; Donald et al., 2001; Pérez et al., 2009). The crystalline regions, primarily amylopectin double helices, reinforce starch's structural integrity (Tester et al., 2004; Copeland et al., 2009; Pérez et al., 2009; Bertoft, 2018).

Starches are examples of food material that soften (gelatinizes) when subjected to heat and moisture due to disruption of their semi-crystalline structure, and solidify again during cooling via retrogradation. Their phase transitions during heating and cooling, alongside associated rheological changes, are crucial for structuring food products. State diagrams have been developed for starch-rich foods like bread (Cuq et al., 2003), crackers (Nikolaidis and Labuza, 1996), biscuits (van der Sman and Renzetti, 2019) and cake (Renzetti, S., van der Sman, 2022) to optimize formulation and processing. A comprehensive understanding of phase transitions, pasting behaviour, and rheological properties across varying starch-to-solvent ratio's can aid in designing optimal food structures and textures.

Starch gelatinization follows structural modifications described by models developed for synthetic side-chain liquid crystalline polymers (Donald, 2001; Waigh et al., 2000). Initially, water penetrates amorphous regions, causing granule swelling (Jenkins and Donald, 1998). Once a critical threshold is reached, stress propagates to crystalline domains, leading to their disintegration. This breakdown includes helix-helix dissociation (from smectic to nematic/isotropic states) and subsequent unwinding of amylopectin double helices (from nematic/isotropic to a gel-like state) (Waigh et al., 2000). The final stage is considered the main melting event of gelatinization.

Differential scanning calorimetry (DSC) has been widely employed to examine how water content affects starch gelatinization. At high water concentrations (>65 % w/w), a single endothermic peak (G endotherm) appears, corresponding to gelatinization and water uptake (Donovan, 1979; Evans and Haisman, 1982; Steeneken and Woortman, 2009). At 35–65 % w/w, an additional endothermic peak (M endotherm) emerges, indicating crystalline melting (Donovan, 1979; Steeneken and Woortman, 2009). At lower hydration levels (<35 % w/w), only a single high-temperature endotherm is observed (Donovan, 1979). These thermal profiles align with the liquid crystalline side-chain model (Waigh et al., 2000). The transition temperature of amylopectin crystal melting varies across starch sources (Vamadevan et al., 2013).

Starch melting transitions influence solubility, density, and viscosity (Roos and Drusch, 2015). In the initial stages, starch granules absorb water and expand until they reach a packed state, causing peak viscosity observed by Rapid Visco Analyzer (RVA) (Balet et al., 2019). Beyond this point, granules start to rupture, dispersing starch molecules into the aqueous phase and reducing viscosity (Copeland et al., 2009). The amorphous phase significantly impacts gelatinization behaviour. The G endotherm in DSC reflects energy changes associated with water absorption and swelling, along with partial amylose leaching (Wang et al., 2014, 2016; Kovrlija and Rondeau-Mouro, 2017). Amylose leaching results from phase separation due to the incompatibility between linear amylose and branched amylopectin (Van Der Sman and Meinders, 2011; Yuryev et al., 1995). Upon cooling, paste viscosity increases due to amylose reassociation, determining final viscosity and setback (Balet et al., 2019).

The thermodynamics for phase transitions (i.e. gelatinization) of glucose homopolymers like starch have been described based on hydrogen bonding using the Flory-Huggins theory for biopolymer melting (van der Sman and Meinders, 2011). The model predicts the state diagram for starch gelatinization (i.e. onset, peak and end temperature of gelatinization) in water solutions considering the volume fraction of water *Φ*_*water*_, the changes in crystallinity during melting ξ, and a composition dependent interaction of starch with water χ. In presence of hydrogen bonding solutes like sugars and soluble fibres, the Flory-Huggins theory has been extended to account for the volumetric density of effective hydrogen bonding sites available in the solvent to interact with starch (van der Sman, 2016; Renzetti, van den Hoek et al., 2021; van der Sman and Mauer, 2019). Notably, the glass transition temperature *T*_*g,s*_ of a dry compound relates to the number of (intermolecular) hydrogen bonds per molecule (Van Der Sman, 2013). The moisture-dependent *T*_*g*_ of a binary mixture of plasticizer with water is a measure of the mixtures hydrogen bond density (Van Der Sman, 2013; van der Sman and Mauer, 2019). Together, these studies indicated that hydrogen bonding largely affects interactions in mixtures of biopolymers, water and sugars. These interactions could also describe the rheological behaviour of carbohydrates solutions. The viscosity of sugars solutions at a specific temprature scaled with the hydrogen bond density (Renzetti et al., 2020; van der Sman and Mauer, 2019), while a master curve was obtained when viscosity was scaled with *T*_*g*_*/T* ratio, with *T* the actual temperature, as it accounts for the hydrogen bond density at temperature *T*. The *T*_*g*_*/T* ratio has been broadly used to describe the rheology of carbohydrates like maltodextrins, sugars, and starch gels (van der Sman et al., 2022).

Against such background, this study aimed to provide a thermodynamic description of phase transitions and a scaling law of rheology after retrogradation of several starches. State diagrams for gelatinization of cereal and tuber starches (wheat, rice, waxy corn, cassava, and potato) were constructed using differential scanning calorimetry combined with Flory-Huggins model for biopolymer melting (van der Sman, 2016). These diagrams were compared to literature data on starch gelatinization in water and sugar solutions to assess their universality in describing phase transitions. Finally, pasting properties and gel rheology were studied at starch concentrations of 5 %, 8 %, and 11 % using RVA and frequency sweeps. The storage (G′) and loss (G″) moduli of starch gels were analyzed against *T*_*g*_*/T* and compared to literature data obtained under similar measurement conditions.

## 2 Materials and methods

### 2.1 Materials

Wheat, rice and potato starch (5 % moisture) were purchased from Sigma-Aldrich (St. Louis, MO, US). Waxy corn starch was provided by Agrana (Wien, Austria). Cassava starch (93.2 % starch, 0.3 % protein and 2.1 % soluble fibres) was supplied by DADTCO (Dutch Agricultural Development and Trading Company, Inhambane, Mozambique).

### 2.2 Methods

#### 2.2.1 Determination apparent amylose

Amylose content was estimated by iodine colorimetry according to (Li et al., 2019) with slight modifications. A standard curve with amylose content ranging from 0 to 100 % was prepared using pure potato amylose (Sigma A0512) and maize amylopectin (Sigma 10,120). Native starches, amylose and amylopectin were suspended in 1 M aqueous NaOH (10 mg/mL), followed by heating in a boiling water bath with shaking. After cooling down to room temperature and five-times dilution in water, a 40 μL aliquot was added into 1 mL water in a 2 mL Eppendorf tube, followed by adding 200 μL iodine solution (0.0025 M I2/0.0065 M KI mixture) and 760 μL water to make up 2 mL solution. The solution was mixed vigorously and then allowed to develop colour for 15 min. The absorbance was read at 600 nm. A standard starch (labelled as 68 % amylose content) of K-AMYL Kit (Megazyme, Ireland) tested with the iodometric assay as reference gave 66.4 ± 0.6 % amylose content at 600 nm. The moisture content of all starches was determined for the calculation of amylose content on a dry weight basis.

#### 2.2.2 Analysis of swelling power

Analyses of swelling power were conducted according to the method of (Konik-rose et al., 2001) with some modifications. Briefly, approximately 40 mg starch were accurately weighed and corrected for moisture to give a dry weight (*S*). The sample was well mixed with water (1 m), vortexed and then held in a water bath equipped with a thermoregulator (Thermomix BU, B. Braun, Germany) for 30 min at 95 °C with gentle shaking. The sample was then cooled to room temperature by being placed in a cool (20 °C) water bath for 5 min. The tube was then centrifuged (10 min, 17,000×*g*). The supernatant was removed by suction and the weight of the residue (B) used to calculate the total starch swelling power:

Starch swelling power = weight of residue (*B*)/dry weight of starch (*S*).

#### 2.2.3 Starch gelatinization in water solution

Starch gelatinization was studied as function of water content as recently described (Renzetti et al., 2021) by preparing different ratio's between starch and water. In particular, starch-water mixtures with water mass fractions ranging from 0.1 to 0.95 (w/w) were studied with incremental steps of 0.1 (w/w) till 0.9.

DSC was used to determine the gelatinization behaviour of starch in the different suspension. High volume hermetic stainless steel cups were filled first with the starch (3 mg–24 mg on dry matter, depending on targeted concentration) and then the solution was added. Cups were closed and stored overnight at room temperature to allow full hydration of the starch. After hydration, samples were then analyzed in a DSC Q200 (TA Instruments, New Castle, USA) by first equilibrating at 10 °C for 5 min and then by heating up at a rate of 5 °C/min to a temperature of: 160 °C for starch concentrations between 10 and 60 %, 180 °C for 70 % starch and 230 °C for starch concentrations of 80 % and above. The onset of starch gelatinization (T_onset_), peak temperature (T_peak_) and end temperature (T_end_) were determined using the analysis tools available in the Universal Analysis software (TA instruments, New Castle, USA). All tests were performed in triplicates.

Data on wheat starch gelatinization were already generated in a previous study (Renzetti et al., 2021) and reported here in comparison to the other starches and to literature data.

#### 2.2.4 Description of starch melting temperature by Flory-Huggins model using the effective number of hydroxyl groups available for intermolecular hydrogen bonding

According to the Flory-Huggins (FH) theory, the biopolymer melting temperature in a water solution can be described as function of the volume fraction of water (*Φ*_*water*_) present in the system, following the equation (van der Sman, 2016):

(1) $$\frac{1}{T_{m}}-\frac{1}{T_{m}^{{}^{\circ}}}=\frac{R}{{\Delta H}_{U}}\frac{v_{U}}{v_{W}}\left[\Phi_{water}-\chi \Phi_{water}^{2}\right]$$

Where $T_{m}$ (K) is the melting temperature of the biopolymer in the system under consideration, $T_{m}^{{}^{\circ}}$ (K) the melting temperature of the dry biopolymer, ${\Delta H}_{U}$ (kJ/mol) is the melting enthalpy per mole of the repeat unit of the biopolymer, $v_{U}$ is the molar volume of the biopolymer repeat unit, $v_{w}$ is the molar volume of water, χ is the FH solvent-biopolymer interaction parameter and *R* is the universal gas constant. It should be noted that in the FH theory, the crystalline phase of biopolymers do not absorb water. The volume fraction of the biopolymer in the rubbery state is computed based on its degree of crystallinity ξ (van der Sman and Meinders, 2011):

(2) $$\Phi_{p}=\frac{\frac{\left(1-\xi \right)y_{p}}{\rho_{p}}}{\frac{y_{w}}{\rho_{w}}+\frac{\left(1-\xi \right)y_{p}}{\rho_{p}}\;}$$

with $y_{w}$ and $y_{p}$ are the mass fraction of water and polymer, respectively, $\rho_{w}$ and $\rho_{p}$ are the mass densities of water and polymer.

For describing the gelatinization behaviour of starch in the sugars and sugar replacers solutions, the volume fraction of water $\Phi_{water}$ in equation (1) is replaced by the effective volume fraction of the solvent, $\Phi_{w,eff}$, comprising the mixture of water and all dissolved sugars according to (van der Sman, 2016):

(3) $$\Phi_{w,eff}=\Phi_{w}+\sum_{i}\Phi_{s,i}\;\frac{\;N_{OH,s}v_{w}}{N_{OH,w}v_{s}\;}$$

where $\Phi_{w}$ is the volume fraction of water, $\Phi_{s,i}$ that of the plasticizer and $v_{w}$ and $v_{s}$ are the molar volume of water and plasticizer, respectively, obtained from the ratio of their molar weight over their mass density. The *N*_*OH,s*_ represents the number of H-bonding sites effectively available within the plasticizer for intermolecular interactions. For water holds *N*_*OH,w*_ = 2. *N*_*OH,s*_ differs from the total number of hydroxyl groups in a molecule as it is corrected for intramolecular hydrogen bond interactions due to stereochemistry (Pawlus et al., 2012). For plasticizers such as sugars and sugar oligomers, *N*_*OH,s*_ is inversely proportional to the glass transition temperature of the pure compound (Van der Sman, 2013):

(4) $$\frac{1}{2}\frac{T_{g\;}-T_{g,w}}{T_{g}^{\infty }-T_{g,w}}=\left(\frac{1}{2}-\frac{N}{N_{OH,s}}\right)$$

Where $T_{g\;}$ is the glass transition temperature of the pure compound, $T_{g,w}$ is the glass transition temperature of pure water (equal to 139 K), $T_{g}^{\infty }$ is the glass transition temperature of a large maltopolymer or starch and equal to 475 K (van der Sman and Mauer, 2019), and $\frac{N}{N_{OH,s}}$ is the inverse of the number of hydroxyl groups per molecule.

For the mixtures under investigation, it holds that $\Phi_{w}+\Phi_{s}=1-\Phi_{p}$ , with $\Phi_{p}$ the volume fraction of starch. The volume fraction of components in the mixtures is computed from the mass fraction using the mass density ρ_i_ of each ingredient, as previously reported (Renzetti et al., 2020).

Following on the description of $\Phi_{w,eff}$, the FH equation (3) is re-written as (van der Sman, 2016):

(5) $$\frac{1}{T_{m}}-\frac{1}{T_{m}^{{}^{\circ}}}=\frac{R}{{\Delta H}_{U}}\frac{v_{U}}{v_{W}}\left[\Phi_{w,eff}-\chi_{eff}{{\cdot \Phi }_{w,eff}}^{2}\right]$$

with the effective interaction parameter equal to (van der Sman, 2016):

(6) $$\chi_{eff}=\chi_{0}+\left(\chi_{1}-\chi_{0}\right){\left(1-\Phi_{w,eff}\right)}^{2}$$

$\chi_{0}$ is the interaction parameter of the hydrated biopolymer and equal to 0.5, while $\chi_{1}$ that of the dry amorphous starch, independently of temperature and equal to 0.8 (van der Sman, 2016). The $\chi_{1}$ parameter is derived from sorption measurements by fitting the obtain moisture sorption isotherm with the Flory-Huggins Free Volume theory described by (van der Sman, 2013; van Der Sman and Meinders, 2011) and holds for the concentrated regime of the biopolymers. In the semi-dilute and dilute regime polysaccharides are prone to aggregation. In amylose solution this is apparent as retrogradation, where chains form junctions via a double helix which are known to absorb hydrophobic compounds in the cavities. Hence, via aggregation (i.e. conformational changes) the more hydrophobic parts of the molecule can be hidden from the water, leading to a change of the interaction parameter (van Der Sman and Meinders, 2011).

#### 2.2.5 Starch gelatinization in solutions of sugar and sugar replacers

The universality of the melting diagram of the studied starches was tested against literature data on starch gelatinization in various sugar solutions. The sugars and sugar replacers for the various starch sources are listed in Table 2, together with their physico-chemical properties and the literature sources. Details on the starch and sugar concentrations used can be found in the specific studies. From the concentrations employed $\Phi_{w,eff}$ was calculated following equation (2) and starch gelatinization data plotted in the state diagrams obtained this study. For wheat starch, the gelatinization temperatures reported by (Allan et al., 2018) for 30 % starch suspension in sugar solutions were compared to those collected in our previous study (Renzetti, van den Hoek et al., 2021).

**Table 1:** Apparent amylose content and swelling power of the different starches. Different letters in the same column indicate statistically significant differences (*p* < 0.05).

Starch	Apparent amylose (%)	Swelling power (g/g)
Cassava	24.4^ab^ ± 5.3	12.7^b^ ± 0.7
Potato	33.4^a^± 10.8	18.3^a^ ± 0.4
Rice	13.5^ab^ ± 2.8	17.1^a^ ± 0.2
Waxy corn	3.6^b^ ± 0.0	10.3^c^ ± 0.9
Wheat	28.6^a^ ± 1.4	14.0^b^ ± 0.6

**Table 2:** Physico-chemical properties of sugars and sugar replacers investigated for the various starches in this study.

Type	M_w_ (g/mol)	ρ_s_ (kg/m^3^)	T_g_ (K)	*N*_*OH,s*_	Starch sources studied with sugar solutions
Arabinose	150	1520	274	3.34	Wheat (Allan et al., 2018)
Ribose	150	1520	262	3.15	Wheat (Allan et al., 2018)
Xylose	150	1520	279	3.43	Wheat (Allan et al., 2018)
Fructose	180	1540	306	3.98	Wheat (Allan et al., 2018); Potato, Rice, Waxy Corn (Allan et al., 2020) Tapioca (Babić et al., 2009)
Galactose	180	1540	305	3.95	Wheat (Allan et al., 2018); Potato, Rice, Waxy Corn (Allan et al., 2020)
Glucose	180	1540	306	3.98	Wheat (Allan et al., 2018); Potato, Rice, Waxy Corn (Allan et al., 2020) Tapioca (Babić et al., 2009) (X. Zhang et al., 2013)
Mannose	180	1540	309	4.05	Wheat (Allan et al., 2018); Potato, Rice, Waxy Corn (Allan et al., 2020)
Tagatose	180	1540	288	3.59	Wheat (Allan et al., 2018)
Isomaltulose	342	1550	334	4.75	Wheat (Allan et al., 2018); Potato, Rice, Waxy Corn (Allan et al., 2020)
Lactose	342	1550	354	5.75	Wheat (Allan et al., 2018)
Maltose	342	1550	353	5.74	Wheat (Allan et al., 2018); Potato, Rice, Waxy Corn (Allan et al., 2020)
Sucrose	342	1550	336	4.48	Wheat (Allan et al., 2018); Potato, Rice, Waxy Corn (Allan et al., 2020); Tapioca (Babić et al., 2009) (X. Zhang et al., 2013)
Trehalose	342	1550	388	7.72	Wheat (Allan et al., 2018); Potato, Rice, Waxy Corn (Allan et al., 2020); Tapioca (Babić et al., 2009)
Maltotriose	504	1550	389	7.81	Wheat (Allan et al., 2018)
Raffinose	504	1550	369	6.35	Wheat (Allan et al., 2018)
Glycerol	92	1261	220	2.36	Wheat (Allan et al., 2018)
Erytritol	122	1450	225	2.75	Wheat (Allan et al., 2018)
Isomalt	344	1550	332	4.69	Wheat (Allan et al., 2018); Potato, Rice, Waxy Corn (Allan et al., 2020)
Maltitol	344	1550	325	4.33	Wheat (Allan et al., 2018); Potato, Rice, Waxy Corn (Allan et al., 2020)
Mannitol	182	1520	266	3.56	Wheat (Allan et al., 2018)
Sorbitol	182	1540	266	3.21	Wheat (Allan et al., 2018); Potato, Rice, Waxy Corn (Allan et al., 2020)
Xylitol	152	1520	249	2.97	Wheat (Allan et al., 2018)

#### 2.2.6 Rapid visco analysis

Pasting behaviour was investigated using a Rapid Visco Analyzer Super 4 (Perten, Hägersten, Sweden). Suspension of samples at concentrations of 5, 8 and 11 % (dry matter weight/weight) were prepared by weighing an appropriate amount of sample to distilled water to reach a total weight of 25 g. The experiment was started with an initial stirring speed of 960 rpm at 50 °C for 60 s. Then, the stirring speed was decreased to 160 rpm while the temperature was increased to 95 °C over 3 min 42 s (rate 12.1 °C per minute), held at 95 °C for 2 min 30 s, decreased to 50 °C over 3 min 48 s (rate 11.8 °C per minute) and held at 50 °C for 5 min. The viscosity was expressed as cP. The RVA parameters were recorded using the TCW3-software for Windows, with pasting onset temperature calculated as the intersection of the baseline before the sudden increase in viscosity and the tangent of the steep viscosity profile after onset. All tests were performed in duplicate.

#### 2.2.7 Small amplitude oscillatory rheology of gels made from starches

Starch suspensions (5, 8 and 11 % w/v) were prepared by dispersing starch in water to reach a final weight of 25 g. Starch gelatinization was performed using RVA as described in previous section. The samples were left at room temperature for 1 h to reach 25 °C. Then the rheological properties of the gelatinized samples were evaluated.

Dynamic viscoelastic properties of gels were determined using a rotary rheometer (Discovery, HR-3, TA instrument Inc., USA) equipped with a parallel plate geometry (40 mm) at 1.0 mm gap. Frequency was set at 1 Hz. Frequency sweeps tests were performed at an angular frequency range of 1–100 rad/s-1 with a strain of 1 %, which was within the linear visco-elastic region (as determined by amplitude sweeps). All tests were performed in triplicates.

#### 2.2.8 T_g_/T of starch gels based on amylose content and starch concentration

The viscosity of water-carbohydrate mixtures has been related to the distance from their glass transitions temperature as described by $T_{g}/T$ (Van Der Sman and Meinders, 2013; van der Sman and Mauer, 2019). Recently, the rheology of starch gels has also been related to $T_{g}/T$ (van der Sman et al., 2022). This study hypothesized that the computed $T_{g}/T$ of the starch gel can describe the gel rheology shortly after gelatinization. During the early stages of granule gelatinization, the outer layer of the granule forms an envelope surrounding the disrupted internal starch polymers. At a critical stress point the swollen envelope ruptures becoming a ghost, releasing the majority of the internal starch molecules, while only a minority of the starch polymers remain trapped by the collapsed ghost (Atkin et al., 1998). At the end of gelatinization the ruptured envelope degrades into ghost remnants. These remnants are derived from the external layers of the granules for a range of starches with different amylose/amylopectin ratios (Atkin et al., 1998). The ghost remnants are composed primarily of amylopectin (Zhang et al., 2014). Under these conditions, it is assumed that amylose primarily governs short-term gelling following gelatinization, while ghost remnants serve as active fillers with elastic properties. Additionally, the water is considered to be equally distributed between amylose and amylopectin within a short time (i.e. 1 h in this study). Amylose and amylopectin showed similar sorption behaviour (Volman et al., 1960), thus indicating the assumption of equal partitioning of moisture may hold. Therefore, the $T_{g}$ of the continuous phase could be estimated based on the amount of apparent amylose present in each gel. The glass transition temperatures of the amylose-water mixtures can be calculated using the Couchman-Karasz equation:

(5) $$T_{g}=\frac{y_{w}T_{g,w}\Delta C_{p,w}+\sum_{i}y_{s,i}T_{g,s,i}\Delta C_{p,s,i}}{y_{w}\Delta C_{p,w}+\sum_{i}y_{s,i}\Delta C_{p,s,i}}$$

with y_w_ the mass fraction of water and y_s,_i the mass fractions of the dissolved plasticizers and leached amylose, ΔC_p,i_ the change in specific heat at the glass transition temperature, and T_g,i_ the glass transition temperature of the pure compound. The glass transition temperature of water is T_g,w_ = 139 K, with ΔC_p,w_ = 1.91 kJ/kg.K (van der Sman, 2016). The value of ΔC_p,s_ is dependent on the class of plasticizers. For monosaccharides and their polymers it holds that ΔCp,s = 0.42 kJ/kg.K. The $T_{g}$ of amorphous starch is 475 K (Van Der Sman and Meinders, 2011). For each starch gel, the $T_{g}$ is calculated using equation (5), (5) with the mass fraction of amylose in the gel estimated based on the starch concentration and the apparent amylose content in each starch source.

#### 2.2.9 Statistical analysis

Analysis of variance (ANOVA) with Tukey's-Test as post-hoc test at a significance level of *p* < 0.05 was performed with SPSS (IBM, version 25, Chicago, US) to determine significant difference among starches in relation to amylose content, swelling, pasting properties and rheology.

## 3 Results and discussion

### 3.1 Apparent amylose content of cereal and tuber starches

The apparent amylose of the investigated starches ranged from 3.6 to 33.4 % (Table 1). Potato and wheat starches showed the highest amylose content while waxy corn the lowest. Rice and cassava were intermediate to those. For cassava, potato, waxy corn and wheat starches the apparent amylose content was well in line with values previously reported by (Sathaporn Srichuwong, 2007). For rice starch the values were well within ranges reported by (Chung et al., 2011; Zhu et al., 2021) for various varieties.

### 3.2 Swelling power of cereal and tuber starches

Waxy corn showed the lowest swelling power while potato and rice starches the highest (Table 1). The swelling power of cassava and wheat were intermediate. The low swelling power of waxy corn starch was in agreement with what earlier reported (Fonseca-Florido et al., 2017). For cassava and potato starches, the swelling power was intermediate to the ranges of 6–25 (g/g) and 6–40 (g/g) at 90 °C, respectively, which have been reported in the literature (Srichuwong et al., 2005; Japheth and John, 2018; Fonseca-Florido et al., 2017). For rice starch and wheat starch, the results of this study fell within the ranges previously reported by (Chung et al., 2011; Zhu et al., 2021) and (Konik-rose et al., 2001), respectively.

### 3.3 Starch gelatinization in water solutions

**Fig. 1:**
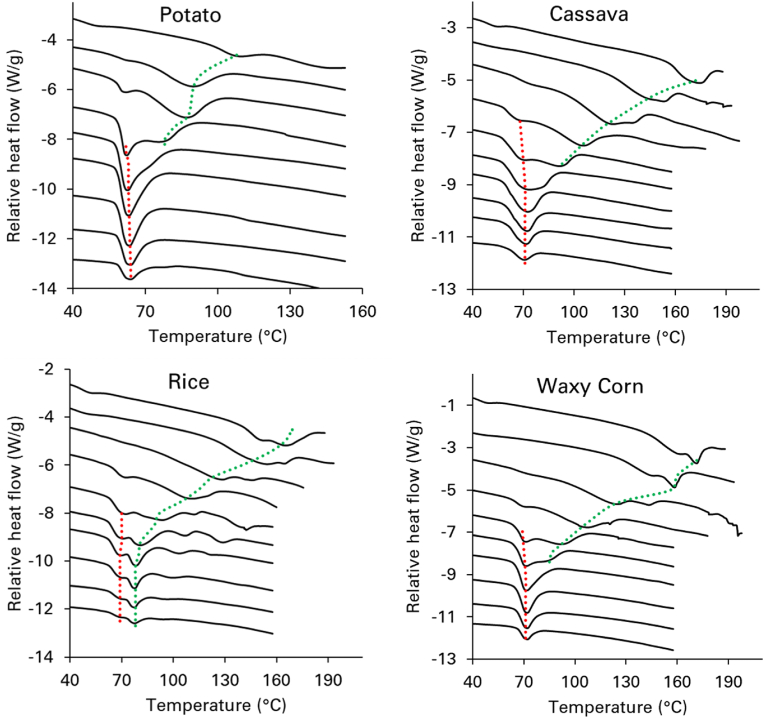
Starch gelatinization profiles from DSC thermograms for various hydration levels. Thermograms are shown from bottom to top from: 0.1, 0.2, 0.3, 0.4, 0.5, 0.6, 0.7, 0.8, 0.9 and 0.95 starch mass fraction. The dotted lines indicate the different endotherms observed: dotted red line = G endotherm of gelatinization accompanied by the uptake of water (Donovan, 1979), dotted green line = M endotherm for melting of crystals *(*Donovan, 1979; Evans and Haisman, 1982).

The process of starch gelatinization was examined under varying hydration levels, with starch mass fractions ranging from 0.1 to 0.95, resulting in distinct DSC thermograms profiles for both cereal and tuber starches (Fig. 1). In dilute solutions (starch mass fraction <0.5), a single starch melting endotherm, referred to as G, was clearly observed, with a peak temperature (T_peak_) between 61 and 77 °C depending on the type of starch. Specifically, at a starch mass fraction of around 0.2 potato starch showed a T_peak_ of 63.6 °C, cassava of 71 °C and waxy corn of 72 °C. For wheat starch the peak was at 61.5 °C as recently shown (Renzetti, van den Hoek et al., 2021). Rice starch showed a slightly different behaviour as a shoulder was observed before the main peak of 77 °C. This initial transition was also labelled as G, in line with the other starches, although a clear peak could not be identified. At starch mass fractions between 0.5 and 0.7, for all starches the DSC thermogram exhibited increased complexity with the broadening of the melting transitions. An additional peak emerged, labelled M, after the initial G endotherm (Fig. 1). When the starch mass fraction increased above 0.7, a single starch melting peak was mainly detected.

**Fig. 2:**
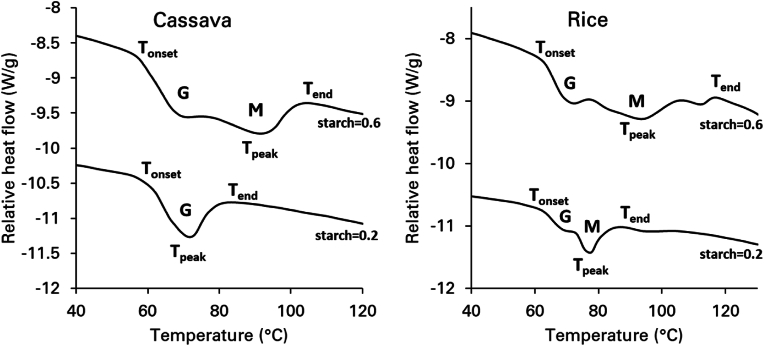
Starch gelatinization profiles for high water contents (i.e. starch mass fraction = 0.2) and intermediate water contents (i.e. starch mass fraction = 0.6). At high water content one main peak is for cassava starch. At intermediate water contents, a broadening of the starch gelatinization peak is observed with the occurrence of two peaks. Based on the description of the different stages of re-organization during starch melting (Waigh et al., 2000), G is defined as smectic/isotropic (or nematic) re-ordering of double helices; M as the main melting event resulting in loss of crystallinity following on the unwinding of double helices via helix/coil transitions. Similar profiles are also observed for potato and waxy corn starch in this study and for wheat starch, as recently reported (Renzetti, van den Hoek et al., 2021). For rice starch, a shoulder before the main peak is already observed at high water contents.

Interpreting the DSC traces at intermediate and low moisture levels is crucial for defining gelatinization parameters, including onset temperature (T_onset_), T_peak_, and end temperature (T_end_). The structural interactions within amylopectin, particularly between its double helices and flexible spacers in the amorphous phase are significantly influenced by water content (Steeneken and Woortman, 2009). This study adopted the two-stage gelatinization model proposed by (Waigh et al., 2000) to analyze DSC data. At high hydration levels (starch mass fraction <0.4), the unwinding of amylopectin double helices can only occur if they first dissociate from their crystallites. As the temperature rises, an endothermic transition is observed, initially due to the gradual dissociation of helices (slow process), followed by a rapid helix-to-coil transition. These transitions appear as a single endotherm (G peak) in the DSC trace, marking the complete loss of helical order. This is evident for cassava starch at mass fraction of 0.2, as shown in Fig. 2 as an example. Similar results were observed for potato and waxy corn starches, and for wheat starch that was already reported in an earlier study (Renzetti, van den Hoek et al., 2021). Under these conditions, T_onset_, T_peak_, and T_end_ were determined from the G endotherm. For rice starch, the G endotherm did not show a clear peak. Therefore, the T_peak_ was always associated with the M endotherm (Fig. 2).

For starch mass fraction between 0.5 and 0.7, as the temperature increases, the thermal energy becomes sufficient to enhance the lateral mobility of amylopectin double helices, leading to a transition from the smectic to the isotropic (for A-type starches) or nematic phase (for B-type starches) (denoted as G in Fig. 2). With further heating, the helices undergo a highly cooperative unwinding process, resulting in the loss of birefringence in the starch granule, which is marked as the M transition. Consequently, the helix-coil transition (M) occurs at a higher temperature than the smectic-to-isotropic (or nematic) phase change (G), producing two major endotherms in the DSC traces. Under these conditions, T_onset_ was determined from the G endotherm, while T_peak_ and T_end_ were derived from the M endotherm (Fig. 2). At very low water levels (starch mass fraction of 0.8 and above), a direct transition from the crystalline state to the helix-coil phase occurs at high temperatures (Waigh et al., 2000). Due to limited backbone and spacer mobility, the formation of a mobile isotropic/nematic phase is not feasible, resulting in a single endotherm in the DSC trace for starch mass fractions of 0.8 and above (Fig. 1). In this case, T_onset_, T_peak_, and T_end_ were all determined from this single endotherm.

**Fig. 3:**
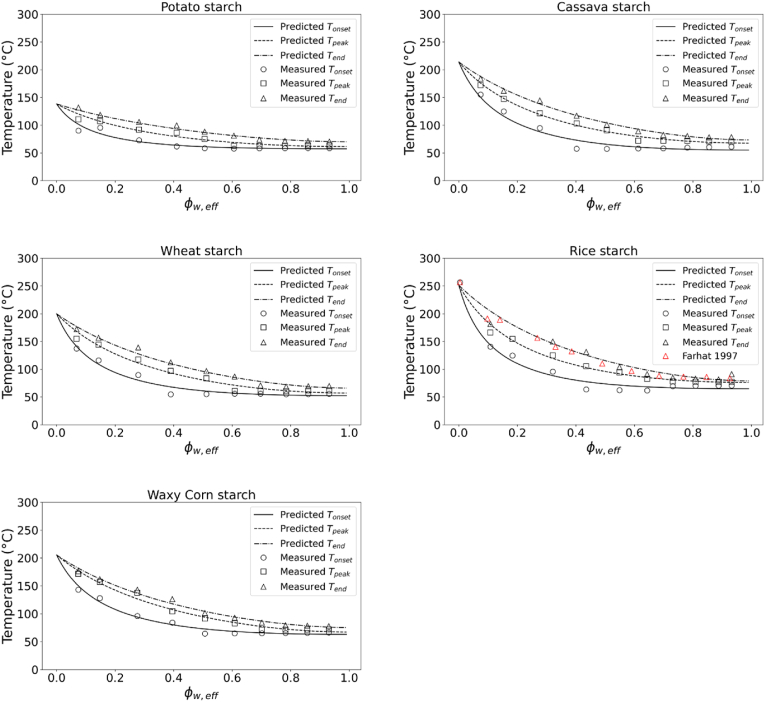
Starch gelatinization profile (i.e. T_onset_, T_peak_ and T_end_) for potato, cassava, wheat, rice and waxy corn starches as described by Φ_w,eff_ at different levels of hydration as obtained by DSC analysis; T_onset_, T_peak_ and T_end_ are obtained from the thermograms based on interpretation of the melting transitions described in Fig. 2; solid and dotted lines show the predicted state diagram of gelatinization following on the application of FH theory for biopolymer melting.

For all starches, the gelatinization behaviour, i.e. T_onset_, T_peak_ and T_end_, as derived from the analysis of DSC thermograms was plotted as a function of $\Phi_{w,eff}$ (which for the water solution is then equal to $\Phi_{water}$; equation (2)) (Fig. 3). T_onset_, T_peak_ and T_end_ data were fitted to the FH theory (equation (1)), with T_m,0_ holding for all three temperatures due to the challenge in determine transition in dry state, but we allowed for differences in ${\Delta H}_{U}$ and degree of crystallinity ξ (Van Der Sman and Meinders, 2011). We expected the degree of crystallinity to decrease from the initial value at room temperature to zero at T_end_, and to be half of the initial value at T_peak_. The coefficients obtained via regression for all starches are shown in Table 3. The FH theory could well describe the gelatinization behaviour of all starches in this study, with the melting enthalpies and degree of crystallinity describing the intrinsic properties of the different starches. The ${\Delta H}_{U,T_{onset}}$ of cassava, rice, waxy corn and wheat starches was in line with the values previously reported by (Farhat and Blanshard, 1997; van Der Sman and Meinders, 2011). The ${\Delta H}_{U,T_{onset}}$ of potato starch was considerably larger than all other starches. A value of 41 kJ/mol has been earlier reported by (Cruz-Orea et al., 2002), which is similar to our results. The trends in the degree of crystallinity are in agreement with the above hypothesis. But, we note that the degree of crystallinity obtained from the FH model are generally higher than what measured by X-ray diffraction (Allan et al., 2020). That may be due to the presence of regions with some degree of ordering which are not captured by X-ray but still contribute to the melting transition (Van Der Sman and Meinders, 2011). Despite the differences in starch source, it is worth nothing that the T_end_ data reported by (Farhat and Blanshard, 1997) for rice starch were well in agreement with the results from this study (Fig. 3).

**Table 3:** Coefficients of the Flory-Huggins model of starch melting obtained via regression for all starches.

Starch	$T_{m}^{{}^{\circ}}$ (K)	${\Delta H}_{U,T_{onset}}$ (kJ/mol)	${\Delta H}_{U,T_{peak}}$ (kJ/mol)	${\Delta H}_{U,T_{end}}$ (kJ/mol)	ξ_onset_	ξ_peak_	ξ_end_
Cassava	490	24.0	26.2	29.0	0.63	0.30	0
Potato	411	40.6	43.2	49.9	0.74	0.34	0
Rice	524	22.9	25.2	25.8	0.70	0.44	0
Waxy corn	478	27.3	28.5	31.0	0.62	0.11	0
Wheat	473	25.1	26.4	28.9	0.66	0.25	0

All coefficients were obtained in this study, except for those of wheat starch that were previously obtained by (Renzetti et al., 2021).

### 3.4 Starch gelatinization in sugar solutions

**Fig. 4:**
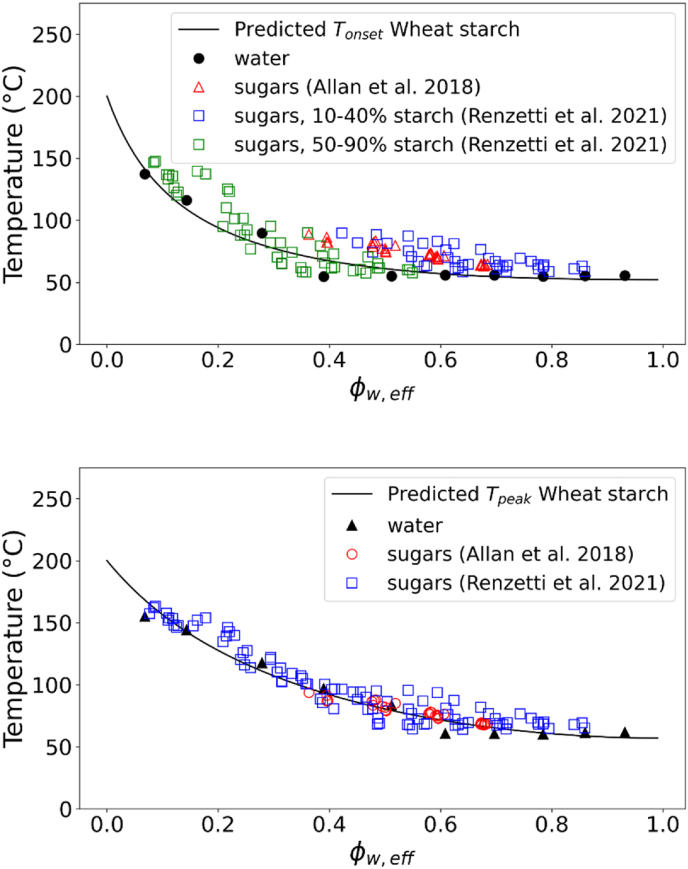
T_onset_ of wheat starch gelatinization showing the experimental data obtained in water and sugar solutions from (Renzetti et al., 2021) at different starch:solution ratio and in sugar solutions at 30 % starch content from Allan et al. (2018) (top). T_peak_ of wheat starch gelatinization showing the experimental data obtained in water and sugar solutions from Renzetti et al. (2021); Allan et al. (2018) (bottom). Solid lines in both figures indicate the predictions from FH theory for biopolymer melting.

**Fig. 5:**
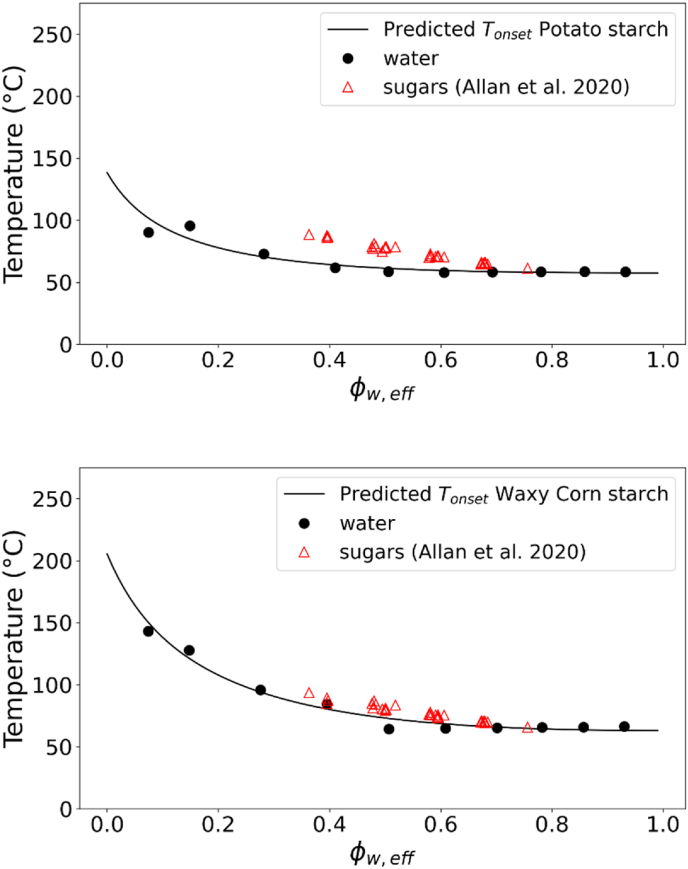
T_onset_ of potato starch gelatinization (top) and of waxy corn starch (bottom) showing the experimental data obtained in water solutions from this study and in sugar solutions from (Allan et al., 2020). Solid lines in both figures indicate the predictions from FH theory for biopolymer melting.

**Fig. 6:**
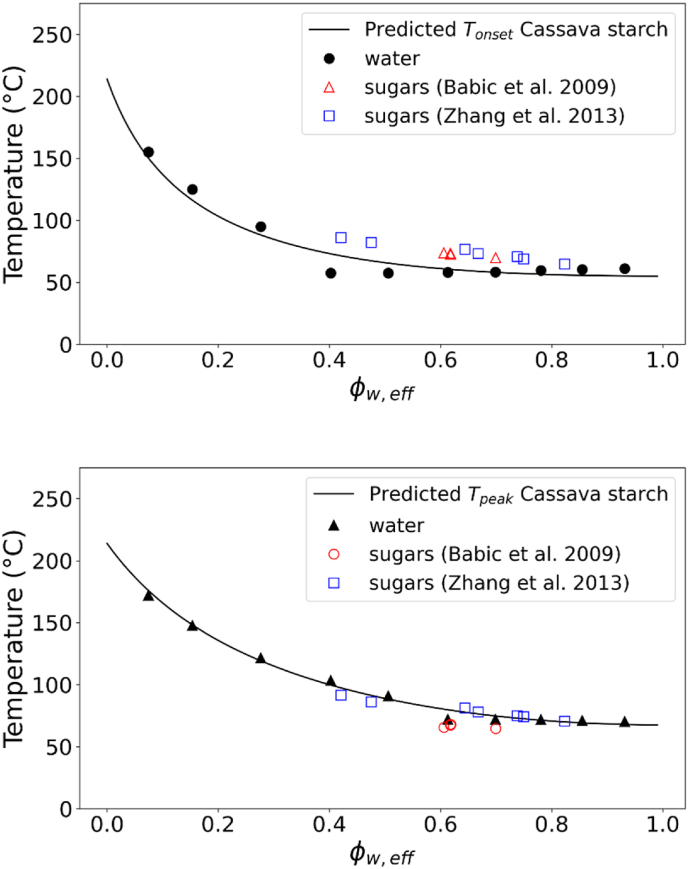
T_onset_ of cassava starch gelatinization (top) and T_peak_ of cassava starch gelatinization (bottom) showing the experimental data obtained in water at different starch:solution ratio from this study and in sugar solutions from (Babić et al., 2009; X. Zhang et al., 2013). Solid lines in both figures indicate the predictions from FH theory for biopolymer melting.

The universality of the gelatinization behaviour described by the state diagrams of Fig. 3 for the different starches was tested against literature data (Table 2). The gelatinization temperatures for potato, waxy corn and wheat starch in presence of sugars obtained from (Allan et al., 2018, 2020) were compared to the state diagram in this study obtained from suspensions in water. For wheat starch, the data were also compared to the sugar solutions from our earlier study (Renzetti, van den Hoek et al., 2021), where several sugar type and concentrations were used at starch concentrations ranging from 10 to 90 %. Allan and co-workers studied starch gelatinization at about 30 % starch concentration in presence of various sugars and polyols. From Fig. 4, it can be observed that our data for 10–40 % suspension of starch in sugars and sugar replacers solutions (Renzetti et al., 2021) matched very well with the data reported by (Allan et al., 2018) for T_onset_ of wheat starch. Deviations from the master curve obtained in sugar solutions can be mainly related to phase separation in sugar-rich and starch-rich phases and the kinetics of sugar ingress in the starch granule (controlled by viscosity), as earlier discussed in depth (Renzetti et al., 2021). The phase separation largely occurs at high to intermediate value of $\Phi_{w,eff}$ (∼0.4–0.7). With increasing starch concentrations and a shift to lower $\Phi_{w,eff}$, the T_onset_ measured in sugar solutions most closely matched the master curve obtained in water solutions. For the T_peak_ of wheat starch gelatinization the data from (Allan et al., 2018) well matched the results from (Renzetti et al., 2021) for gelatinization in water and in sugar solutions (Fig. 4). Contrary to the onset, the peak temperature of gelatinization is mainly controlled by thermodynamics (Renzetti et al., 2021), thus explaining the good agreement with the FH theory (i.e. chi-squared for T_peak_ between experimental and model predictions were lower than for T_onset_). Also the data for the T_onset_ of potato starch and of waxy corn starch in sugars solutions from (Allan et al., 2020) well matched the results of this study (Fig. 5). Furthermore, the T_onset_ and T_peak_ of cassava starch in water and sugar solutions reported by (Babić et al., 2009; X. Zhang et al., 2013) was also well in agreement with our state diagram (Fig. 6). The agreement between the data in this study on gelatinization of five different starches and those reported by other groups (Allan et al., 2018, 2020; Babić et al., 2009; Farhat and Blanshard, 1997; Zhang et al., 2013) is remarkable considering the differences in starch sources and methodological approaches. Overall, Fig. 3, Fig. 4, Fig. 5, Fig. 6 indicate that our approach could be universally applied to starch from any botanical source mixed with hydrogen-bonding plasticizers (like water and/or sugars). Starches were discriminated by degree of crystallinity, the melting temperature of the dry biopolymer $T_{m}^{{}^{\circ}}$, and the melting enthalpy per mole of the repeat unit of the biopolymer ${\Delta H}_{U}$. Amylose content appears not to be of importance for the phase transitions, but it will govern the gel formation upon cooling after the gelatinization phase transition.

### 3.5 Pasting properties of starches at different concentrations

**Fig. 7:**
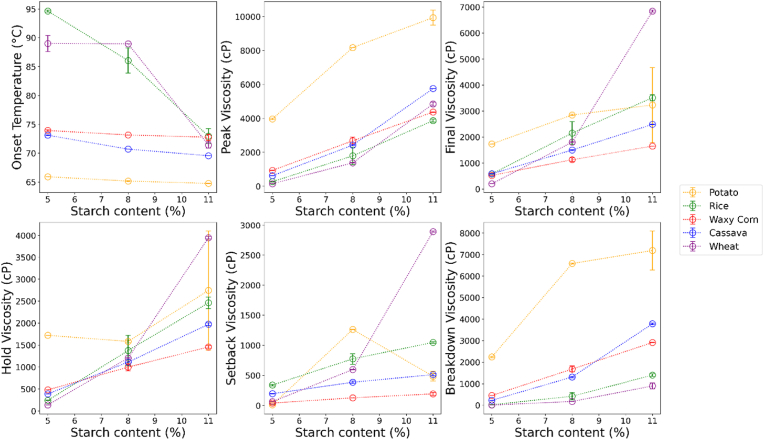
RVA parameters obtained for the different starches plotted as function of starch concentration in the suspension.

The pasting parameters obtained for the different starches at 5, 8 and 11 % are shown in Fig. 7. At 5 % concentration potato starch showed the lowest pasting onset temperature while rice and wheat starch the highest (p < 0.05). With increasing concentration, wheat and rice starch showed a linear reduction in pasting onset temperature, reaching similar values as waxy corn at the highest concentration. At 11 % concentration, potato starch was still the one with lowest pasting onset temperature, followed by cassava (p < 0.05). At low starch concentration, the pasting onset temperature is affected by several factors related to granule morphology and molecular properties (i.e. amylose:amylopectin ratio, amylose-lipid complexes, phosphorous content) (Balet et al., 2019). With increasing starch concentration above 10 %, swelling becomes a limiting factor since a space filling gel is formed and the granules cannot swell to their maximum (Van Der Sman, 2018). These mechanisms explain the reduction observed in pasting onset temperatures. In agreement with these findings, (Mauro et al., 2023) observed a sharp reduction in pasting onset temperature for wheat and rice with increasing starch concentration, while the reduction was rather small for potato, tapioca and waxy corn.

The peak viscosities of 5 % starches showed significant differences among all of them, with potato starch having the highest viscosity and wheat the lowest (p < 0.05). With increasing concentration, wheat starch showed a significant increase in peak viscosity compared to the other starches, resulting in a peak viscosity higher than rice and similar to waxy corn at 11 %. At the highest concentration tested, potato starch remained the one with highest viscosity, followed by cassava (*p* < 0.05). At 5 %, all starches differed significantly for hold viscosity, while at 11 % only waxy corn and wheat differed with each other (*p* < 0.05). The peak viscosity of starch suspension is related to granule rigidity and resistance to breaking during heating and shearing. The peak is achieved when the rate of swelling equals the rate of breaking down (Balet et al., 2019). Potato and cassava starches have typically larger granule size and longer branched amylopectin chains than cereal starches (Hoover, 2001; Singh et al., 2003), resulting in higher peak viscosities over concentration ranges from 2 to 11 % (Mauro et al., 2023).

At 5 % starch concentration, the breakdown showed significant differences among all samples with potato starch having highest paste breakdown and wheat starch the lowest (*p* < 0.05). At 11 % potato starch still had the highest breakdown, wheat and rice the lowest, while cassava and waxy corn were intermediate (*p* < 0.05). Breakdown viscosity is a measure of the ease of disintegration of swollen starch granules. A high swelling of starch, also associated with high peak viscosity, makes the starch more susceptible to disintegration. High breakdown is typically observed in potato starches (Wiesenborn et al., 1994) and affected by the high phosphorus content (Noda et al., 2006). In agreement with our results, potato and cassava showed higher breakdown than wheat and rice (Mauro et al., 2023).

With regards to the final viscosity, wheat starch showed the sharpest increase as function of concentration, being the lowest at 5 % while becoming the highest at 11 % (*p* < 0.05). At the highest concentration, all other starches showed similar final viscosity. The sharp increase in final viscosity for wheat starch was also reflected in the setback, as wheat also had the highest value at 11 %, while waxy corn the lowest. On the contrary, rice showed the highest setback at 5 % and potato the lowest (*p* < 0.05). The setback and final viscosity indicate the tendency of the leached amylose to re-associate with decrease in temperature (Kaur et al., 2007). Amylose content and amylopectin chain distribution affect both setback and final viscosity (Li et al., 2020), with cereal starches showing a higher rate of increase in these viscosity parameters with increasing concentration than potato and cassava (Mauro et al., 2023).

### 3.6 Frequency sweeps of starch gels as function of concentration

**Fig. 8:**
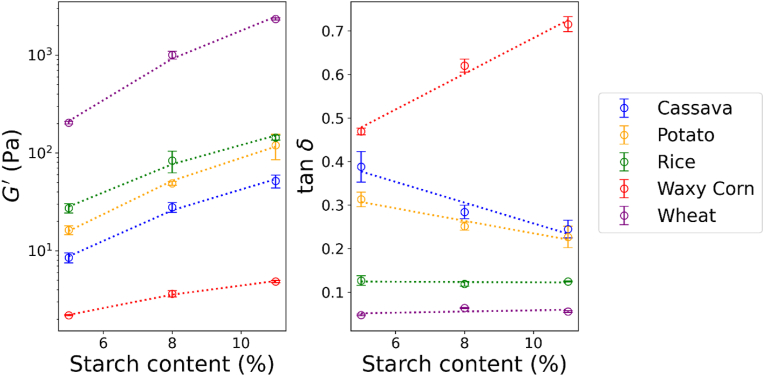
G′ and tan δ obtained at 1 rad/s from frequency sweeps plotted as function of starch content. Error bars represent the standard deviations (*n* = 3). The dotted lines represent the model fitting using a power law and a linear function for G′ and tan δ, respectively.

The frequency sweeps of gels were obtained for all starches at 5, 8 and 11 % concentration (Supplementary Fig. S1). For all samples, G′ and G″ increased with increasing starch concentration. However, differences in the absolute values of G′ and G″ and in their relative ratio could be observed among starches. Wheat starch showed the highest ranges of G′, while waxy corn the lowest. For wheat and rice starches, the frequency sweep tests showed near independence of frequency within the frequency range, indicating a well-formed gel structure. The G′ and tan δ extracted at a frequency of 1 rad/s were plotted as function of starch content (Fig. 8). Regardless the concentration, wheat starch showed the highest G′ and waxy corn the lowest, with cassava, potato and rice being intermediate among these two. For all starches, the increase in G′ was a power law function of the concentration (R^2^ > 0.99). In contrast, the tan δ (i.e. the ratio of G″ over G’) showed different trends depending on the starch source. Potato and cassava starch showed a linear reduction in tan δ with increasing starch content, indicating an enhancement of the elastic moduli over the viscous one. The opposite trend was observed for waxy corn starch with a linear increase in tan δ. Instead, no changes were observed for wheat and rice starches.

The observed differences in the rheology of the starch gels after 1 h cooling may be first attributed to differences in the apparent amylose content in the gels. When starch granules are heated in water to their gelatinization temperature, the crystalline structure within the granules breaks down, allowing the granules to swell as they absorb water (Jenkins and Donald, 1998; Waigh et al., 2000). Simultaneously, amylose molecules inside the granules leach out. At the end of the gelatinization process, the majority of the internal starch molecules are released from the ruptured granules, leaving a continuous phase rich amylose and ghost remnants mainly composed of amylopectin (Atkin et al., 1998; Zhang et al., 2014). This amylose is the dominant gel-forming component in gelatinized starch, due to its ability to form networks with water through intermolecular hydrogen bonding (Hennig et al., 1976; McGrane et al., 2004), embedding the ghost remnants in a continuous matrix (Ring, 1985; Ring and Stainsby, 1982; Wong and Lelievre, 1981). The short-term development of gel structure and crystallinity in starch gels is dominated by gelation of the solubilized amylose (Miles et al., 1985). Long-term increases in the modulus of starch gels are linked to a reversible crystallization, involving amylopectin, within the granules on storage. The granule remnants are amylopectin-enriched structures and could be described as swollen hydrated polymer composites that behave as active filler particles reinforcing the continuous matrix of entangled molecules (Biliaderis, 1998; Liu and Lelievre, 1992; Rao et al., 1997; S. G. Ring, 1987). This suggests that the amylose-rich continuous phase is critical to gel rheology, with elastic ghost remnants reinforcing the continuous amylose network (Fannon and Bemiller, 1992; Svegmark and Hermansson, 1991).

**Fig. 9:**
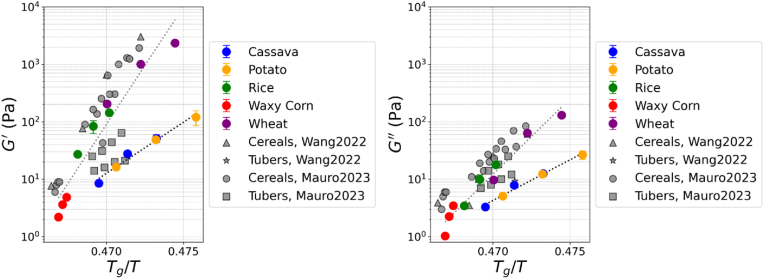
G′ and G″ from frequency sweeps plotted as function of *T*_*g*_*/T* computed from amylose concentration in the gels. Relevant literature data are also plotted for comparison with the results of this study. The dotted lines represent data fitting using a power law equation.

In order to test the influence of amylose content on the rheology of the gels, the apparent amylose content in the gels was calculated for each starch type and concentration. It should be also noted that the rheology of starch gels scales with *T*_*g*_*/T* (van der Sman et al., 2022). The *T*_*g*_ of gelatinized starch can be mainly attributed to the bulk amorphous phase from amylose (Biliaderis et al., 1986), which for starches is reported to 475 K regardless of the specific source (Van Der Sman and Meinders, 2011). This hypothesis assumes that the water is equally distributed between amylose and amylopectin within a short time (i.e. 1 h in this study). The partitioning of water between the two biopolymers is controlled by their sorption properties, which for amylose and amylopectin were reported to be similar (Volman et al., 1960). Considering the assumption of equal partitioning of moisture may hold, the G′ and G″ of the starch gels are shown to be a function of *T*_*g*_*/T*, with application of the Couchman-Karazs equation (Fig. 9). Both G′ and G″ scaled with *T*_*g*_*/T*, although the G′ and G″ values were higher for the cereals than the tubers. Recent studies from (Mauro et al., 2023; Wang et al., 2022) also reported the rheology of starches from different sources in conditions of gelling (within 2 h of heating and cooling to 25 °C) similar to this study. Wang and co-workers (Wang et al., 2022) investigated corn starches with different amylose contents and a tapioca starch. Mauro and co-workers (Mauro et al., 2023) studied wheat, corn, waxy corn, and rice among the cereals, while potato and tapioca among the tubers. Despite the differences in the specific sources of starch and testing conditions, the data from these studies well aligned with our results, albeit the values of G′ and G″ were generally higher than ours. In agreement with these results, an increase in G′ and G″ with increasing amylose content was also observed for potato starches (Ortega-Ojeda et al., 2004), corn (Cheng et al., 2024), rice (Zhong et al., 2022) and wheat (Song et al., 2024). Differences in the scales of G′ and G″ between cereals and tubers may be attributed to structural and compositional differences. Remnants of the starch granules strongly influence the rheological behaviour of the starch gel (Ortega-Ojeda et al., 2004) since the swollen starch granules form a close-packed gel structure (Svegmark and Hermansson, 1993, 1990, 1991). Thus, particle rigidity, integrity of the swollen granules and the extent of swelling can contribute rheological differences (Li and Yeh, 2001). Additionally, differences in amylopectin structure among the starch sources may also explain the gelling properties. Long amylopectin chains promotes the formation of starch gels with higher G′ and G″ than those with short chains (Lu et al., 2009; Mizukami and Takeda, 2000; Song et al., 2024). Therefore, differences in amylopectin chain distribution and its effect on the rate of retrogradation (Zhu and Liu, 2020) could be an additional factor for the distinct trends observed in the rheology of cereal and tuber starches.

## 4 Conclusion

In this study, we constructed detailed melting diagrams for five starches from both cereal and tuber sources, capturing onset, peak, and end gelatinization temperatures. These transitions were successfully modeled using the Flory-Huggins theory for polymer melting, extending its applicability beyond prior single-starch systems, i.e. wheat (Renzetti, van den Hoek et al., 2021; van der Sman and Mauer, 2019). Notably, the diagrams also successfully captured melting transitions in the presence of sugars by treating water-sugar mixtures in terms of effective solvent volume fraction ($\Phi_{w,eff}$), which represents the volumetric density of hydrogen bonding sites available for interactions with starch. This conceptual framework enabled us to collapse disparate datasets—both our own and from previous literature—onto a common melting curve, despite variations in experimental methodology and starch origin. This represents, to the best of our knowledge, the first comprehensive demonstration of the Flory-Huggins model's ability to universally describe melting diagrams across a range of botanical starch sources in both aqueous and sugar-rich environments. Notably, systematic deviations from model predictions for T_onset_ were observed at high sugar concentrations, consistent with phase separation phenomena that likely emerge at both intermediate and excess solvent conditions. These deviations echo trends previously observed for wheat starch (Renzetti, van den Hoek et al., 2021), but are here shown to be a generalizable feature of complex starch-solvent interactions.

Building on this thermodynamic foundation, we further investigated the rheological behaviour of gelatinized starches. Drawing on the established relationship between hydrogen bonding, solvent composition, and the moisture-dependent glass transition temperature *T*_*g*_ (Van Der Sman, 2013; van der Sman et al., 2022), we hypothesized that the viscoelastic properties of these gels scale with the *T*_*g*_/*T* ratio—that is, the ratio of glass transition temperature to measurement temperature. Importantly, this *T*_*g*_/*T* value was not measured directly, but was calculated based on the apparent amylose concentration. Assuming amylose as the dominant gel-forming component and equal partitioning of water between amylose and amylopectin, we found that both storage (G′) and loss (G″) moduli across starch concentrations could be quantitatively predicted by this calculated *T*_*g*_/*T* ratio. Remarkably, this *T*_*g*_/*T*-based scaling held true not only for our own dataset but also for rheological data from other studies conducted under similar conditions. Importantly, we observed distinct scaling laws for cereal and tuber starches, revealing fundamental differences likely linked to amylopectin architecture, granule morphology, rigidity, and minor component content (e.g., protein and minerals).

Taken together, these findings advance the current understanding of starch gelatinization and gelation by introducing a unifying thermodynamic and rheological framework applicable to a broad range of starch types and solvent systems. This dual-approach—combining melting diagrams and *T*_*g*_/*T*-based rheology—offers a powerful new framework for the design and optimization of starch-based food products.

## CRediT authorship contribution statement

**Stefano Renzetti:** Conceptualization, Methodology, Formal analysis, Visualization, Supervision, Writing – original draft, Writing – review & editing, Funding acquisition. **Jolanda Henket:** Investigation, Data curation. **Eric Raaijmakers:** Investigation, Data curation. **Irene van den Hoek:** Investigation, Data curation. **Ruud van der Sman:** Methodology, Writing – review & editing.

## Declaration of competing interest

Authors declare there is no conflict of interest.

## Data Availability

Data will be made available on request.
