# Supplementary material for: Hydrogen bond density and glass-transition temperature govern gelatinization and gel rheology in cereal and tuber starches
Source: Curr Res Food Sci. 2025 May 29;10:101101. doi: 10.1016/j.crfs.2025.101101 (PMC12167104; doi:10.1016/j.crfs.2025.101101)
Supplement: Multimedia component 1 [file mmc1.docx]

*
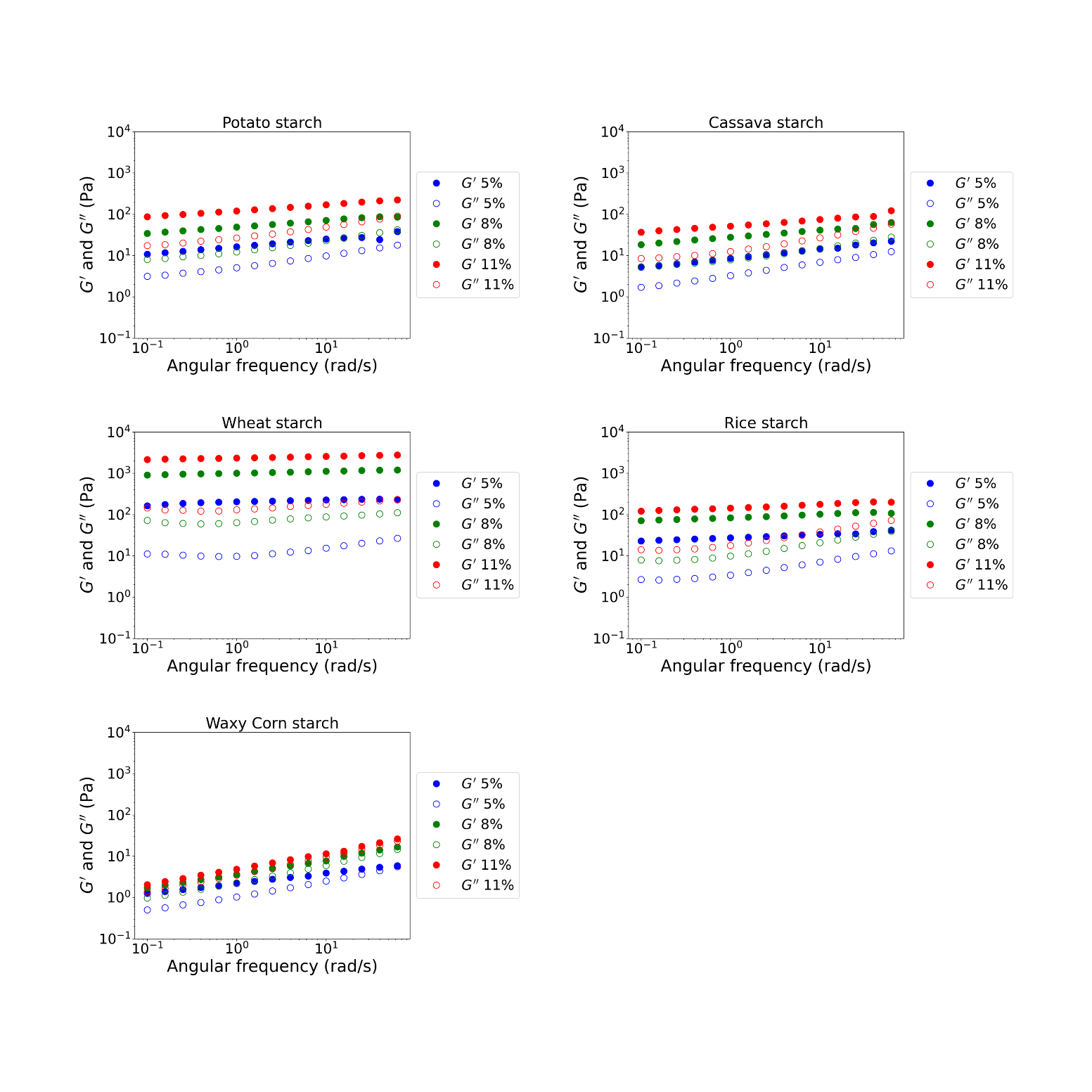
*

Figure S1. G’ and G’’ obtained from frequency sweeps for the different starches at concentrations of 5, 8 and 11%. Measurements were perfromed from samples obtained from RVA after one hour cooling to 25 °C.
